# Hon-yaku: a biology-driven Bayesian methodology for identifying translation initiation sites in prokaryotes

**DOI:** 10.1186/1471-2105-8-47

**Published:** 2007-02-08

**Authors:** Yuko Makita, Michiel JL de Hoon, Antoine Danchin

**Affiliations:** 1 Unit of Genetics of Bacterial Genomes, Institut Pasteur, URA CNRS 2171, 28 rue du Docteur Roux, 75724 Cedex 15, Paris, France; 2 Center for Computational Biology and Bioinformatics, Columbia University, 1130 St Nicholas Avenue, New York, NY 10032, USA; 3 Genomic Sciences Center, RIKEN, 1-7-22, Suehiro, Tsurumi-ku, Yokohama, Kanagawa 230-0045, Japan

## Abstract

**Background:**

Computational prediction methods are currently used to identify genes in prokaryote genomes. However, identification of the correct translation initiation sites remains a difficult task. Accurate translation initiation sites (TISs) are important not only for the annotation of unknown proteins but also for the prediction of operons, promoters, and small non-coding RNA genes, as this typically makes use of the intergenic distance. A further problem is that most existing methods are optimized for *Escherichia coli *data sets; applying these methods to newly sequenced bacterial genomes may not result in an equivalent level of accuracy.

**Results:**

Based on a biological representation of the translation process, we applied Bayesian statistics to create a score function for predicting translation initiation sites. In contrast to existing programs, our combination of methods uses supervised learning to optimally use the set of known translation initiation sites. We combined the Ribosome Binding Site (RBS) sequence, the distance between the translation initiation site and the RBS sequence, the base composition of the start codon, the nucleotide composition (A-rich sequences) following start codons, and the expected distribution of the protein length in a Bayesian scoring function. To further increase the prediction accuracy, we also took into account the operon orientation. The outcome of the procedure achieved a prediction accuracy of 93.2% in 858 *E. coli *genes from the EcoGene data set and 92.7% accuracy in a data set of 1243 *Bacillus subtilis *'non-y' genes. We confirmed the performance in the GC-rich Gamma-Proteobacteria *Herminiimonas arsenicoxydans, Pseudomonas aeruginosa*, and *Burkholderia pseudomallei *K96243.

**Conclusion:**

Hon-yaku, being based on a careful choice of elements important in translation, improved the prediction accuracy in *B. subtilis *data sets and other bacteria except for *E. coli*. We believe that most remaining mispredictions are due to atypical ribosomal binding sequences used in specific translation control processes, or likely errors in the training data sets.

## Background

Genome sequencing provides investigators with a plain genome text, with no biological indication of the genes' location. The first task associated with genome annotation is therefore gene identification. In recent years, gene prediction methods have been developed as part of many genome projects. Based on criteria strictly defined by previously known genes, the best computational gene identification methods for prokaryote genomes show sensitivities of 98–99% or higher for proper identification of the genes' reading frames [1]. However, based on the widespread assumption that Open Reading Frames (ORFs) and Coding DNA sequences (CDSs) label the same objects, this level of prediction accuracy is calculated using the 3' end location of each gene, not the actual gene span. One of the most widely used methods, Glimmer [1], tends to predict the CDS to be the longest possible ORF displaying a particular nucleotide pattern based on Markov chain analysis and starting with the first possible translation initiation codon (ATG, TTG or GTG). The conceptual basis of Glimmer rests on the original periodical Markov Chain Analysis approach, GeneMark, which for precise prediction of the gene's 5' end, also considers sequence features located upstream of the translation initiation sites. The resulting accuracy is 5–30% lower than the 3' end predictions [2]. GeneMark often succeeds better in correct gene identification because it is based on discrimination between typical protein coding states and atypical protein coding states, which is assumed to be populated with genes horizontally transferred into a given microbial genome. This was illustrated, for example, with identification of the *cyaY *gene in *Escherichia coli *[3] and the *secE *gene in *Helicobacter pylori *[4].

A more accurate translation initiation site (TIS) prediction is important not only for the annotation of unknown CDSs but also for operon prediction [5] and promoter prediction. Furthermore, in silico prediction of genes coding for small untranslated RNAs [6] also depends on the correct identification of intergenic (inter CDS) distances.

Most existing tools use an unsupervised learning method, using *E. coli *data sets for validation, due to the lack of experimentally validated data sets in other organisms. In the present work, we adopted a supervised machine learning method for the following reasons. First, we took into account that in the current annotation situation, human annotation is still more reliable than any computational genome-wide predictions, suggesting that by trying to mimic the human approach we might construct more reliable data sets. Second, supervised learning assumes that we implement some knowledge of what we can consider as the most important elements in the prediction method. Furthermore, it is difficult to know the range of correct applicability with unsupervised algorithms without deep knowledge of the algorithms. For example, in a recent comparison between the TiCo algorithm and MED-Start, the latter showed surprisingly low accuracies (around 5%) with high GC-content genomes, although it showed over 90% accuracy in the E. coli data set [7]. This is in line with the general difficulty to identify translation start sites in GC-rich organisms where the lack of A or T nucleotides results in long ORFs due to purely statistical reasons. To construct an in silico model of translation initiation based on biological knowledge, we take into account the following elements.

First of all, the Ribosome Binding Site (RBS, also named the Shine-Dalgarno sequence, after the name of the authors who proposed that mRNA had to interact with the 16S RNA to permit initiation of translation [8]) is one of the most important elements for translation initiation. The RBS sequence is recognized by a sequence near the 3' end of 16S rRNA in the 30S ribosomal subunit. After the 30S ribosomal subunit binds to mRNA by base pairing to the RBS sequence, the fMet-tRNA identifies the initiation codon and binds to the complex. Next, the 50S ribosomal subunit binds to the complex and begins to elongate the nascent polypeptide [9].

Compared to *Bacillus subtilis*, *Escherichia coli *has relatively short or poorly conserved RBS sequences. To be able to separate these weak RBS sequences from the noise, *E. coli *has an S1 protein that plays an important role in the correct presentation of most mRNAs to the ribosome. The recognition signal of the S1 protein for binding mRNA has been studied in its molecular details but is not yet completely understood. The S1 protein binds to the leader sequence of mRNAs, upstream of the RBS sequence. On synthetic RNAs, S1 has no strict sequence specificity and binds polyU, polyC, and polyA, as well as various heterogeneous RNAs. However, it has been shown to present sequences possessing the GAGG sequence to the RegB nuclease of bacteriophage T4 [10], indicating that it has indeed a role in the recognition of the core sequence of the RBS. In contrast, *B. subtilis *or A+T-rich Firmicutes do not possess an S1 protein. (*B. subtilis *has a counterpart, YpfD, but this protein is not involved in translation [11]). Finally, both *E. coli *and *B. subtilis *are weakly AU-rich upstream of the RBS sequence. A difficulty encountered with GC-rich organisms is that long Gs stretches can easily be mistaken for authentic RBSs. For an accurate prediction of the TIS, we also need to consider translational reinitiation when several cistrons belong to a common transcript. Translational reinitiation frequently occurs if the initiation codon is an AUG, a RBS sequence is present, and the termination codon of the preceding CDS lies between the RBS sequence and the AUG or overlaps the RBS. In this case, the 70S ribosome does not need to be dissociated into 50S and 30S ribosome subunits [9] to allow translation initiation. Therefore, translational reinitiation signals may be different from canonical initiation.

The RBS sequence is usually located 3–8 nt upstream of the start codon. The optimal spacing depends on exactly which bases at the 3' end of 16S rRNA participate in the interaction. The start codon is preferably AUG. Weaker base pairings with fMet-tRNA to initiation codons are less efficient for translation initiation [12]. The preference for alternative start codons varies between species. *B. subtilis *prefers UUG rather than GUG, while the opposite is true for *E. coli *(Table 1). The selection ratio of the primary AUG in *E. coli *is higher than in *B. subtilis*, and this is one of the reasons making that standard prediction accuracy for *B. subtilis *is lower than for *E. coli*, in spite of the "stronger" RBS sequence.

**Table 1:** Frequency of translation initiation site code

Organism	Location	ATG	GTG	TTG
*E. coli*	True position	**90.9%**	7.2%	1.9%
	Upstream	36.3%	26.1%	**37.6%**
	Downstream	40.7%	**43.4%**	15.9%

*B. subtilis*	True position	**80.7%**	8.6%	10.7%
	Upstream	**35.9%**	31.6%	32.5%
	Downstream	**44.4%**	30.9%	24.7%

An A-rich sequence following the start codon is typically found in both *B. subtilis *and *E. coli *[13]. Those A-rich (A/U rich) sequences probably stimulate translation initiation by excluding secondary RNA structures [14].

Furthermore, we also took into account the fact that biases introduced by translation may affect the translation process, discriminating between two types of intergenic distance distributions; head to head (< -- >) and tail to head (- > - >) cases, for assuming the non-operon/operon structures.

For each of these biological considerations, we assessed to what degree they can contribute to the TIS prediction accuracy, as described in the Results. Based on this evaluation, we selected six elements (see Methods) and combined them into a single score function using Bayesian statistics.

This Bayesian supervised learning method for TIS prediction, which we named Hon-yaku ("translation" in Japanese), showed a prediction accuracy of over 90% for both *E. coli *and *B. subtilis*. We also applied this method to GC-rich Gamma-Proteobacteria that do not have any experimentally validated TIS data sets. Our Python scripts can be downloaded [15]. After construction of a reference data set based on core genome sequences, the scripts can be used with some basic knowledge of Python to predict TISs in newly sequenced bacterial genomes. To obtain training data sets, we chose genes that have strong sequence similarity to *E. coli *or *B. subtilis *data sets, retaining the genes that display genome persistence [16]. Our algorithm also performed well in *P. aeruginosa, B. pseudomallei*, and the newly sequenced genome of the Beta-proteobacterium *Herminiimonas arsenicoxydans*, which can metabolize arsenic.

## Results and discussion

### RBS sequence motif comparison

Except for some special cases such as leaderless genes, most genes have an RBS sequence around 3–8 bp upstream from the TIS. We considered several RBS motif categories that represent the gene essentiality, the position of each operon, and the organism specificity.

The first gene of an operon typically has a longer intergenic space to the previous gene than subsequent genes. By contrast, the RBS sequences of subsequent genes often overlap with the coding region of the previous gene. In these latter cases, the RBS sequence is influenced by the coding sequence. We constructed a data set of overlapping motifs and a data set of non-overlapping motifs to assess the effect of codon usage on RBS sequence. We used the sequenced 30 bp upstream and 20 bp downstream from the TIS to calculate an information content (IC) score (Eq. 1). We constructed a data set of overlapping motifs and a data set of non-overlapping motifs to assess the effect of codon usage on RBS sequence. We used the sequenced 30 bp upstream and 20 bp downstream from the TIS to calculate an information content (IC) score (Eq. 1). The IC scores for RBS sequences overlapping CDSs (IC = 12.4) were slightly smaller than for non-overlapping RBS motifs (IC = 12.9) (Table 2). The difference is not due to a variation in the RBS sequence itself but to a difference in the A nucleotide content of the sequences upstream from the RBS. The IC score of the RBS sequence (AGGAG) was almost identical in both cases (IC = 4.7, and IC = 4.6, respectively). The lack of conservation of A-rich sequences when CDSs and RBSs overlap is likely due to constraints specific to translation reinitiation [9]. In this case, the mRNA is already bound to the ribosome, permitting to relax the constraints needed for translation initiation site selection, while allowing to accommodate overlap with the protein reading frame.

**Table 2:** Comparison of information content score in various data sets

Organism	Data set	# of genes	Score of IC	Reference
*E. coli*	EcoGene	858	12.9	Rudd K.E. [37]
	Overlapping	120	12.4	Methods
	Non-overlapping	205	12.9	Methods
	Essential	153	12.3	Fang G. *et al*. [16]
	Persistent	309	12.4	Fang G. *et al*. [16]

*B. subtilis*	non-y	1243	**16.3**	Yada T. *et al*. [38]

Currently, essential genes are defined by in vivo experiments in several species [17-19]. To investigate a possible contribution of gene essentiality to RBS sequence conservation, we calculated the IC for essential genes and persistent genes, which are strongly conserved in most bacterial genomes [16]. Interestingly, we could not detect specific RBS sequence features which would relate to gene essentiality or persistence, thus validating the use of persistent genes in the training set (as they would not introduce a bias in TIS identification). The IC scores of these particular sets were not larger than the EcoGene data set score, which is the largest data set. We therefore decided to use the RBS sequences extracted from the EcoGene data set.

By contrast, there are significant differences between organisms: *B. subtilis*, which does not have a S1 protein, shows the largest score of the three organisms (Table 2). This is consistent with the role of protein S1 in the attachment of the mRNA to the 16S rRNA in *E. coli *[20].

### Accuracy of the method

#### Selecting the order of the Markov model

We used a Markov model to score the relevant DNA sequences near the TIS. If the training data set is sufficiently large, a higher order model may provide a better description of the motif. We examined the accuracy for a 0th, 1st, and 2nd order Markov model in a leave-one-out cross validation analysis (Table 3). The 0th order Markov model showed the highest accuracy in *H. arsenicoxydans*, which has the smallest sample of training data, while the 1st order Markov model was best for *E. coli *and *B. subtilis*. Moreover, although we had over 1200 instances in the training data set of *B. subtilis*, the 1st order Markov model gave a better accuracy than the 2nd order Markov model due to many similar instances in the data set.

**Table 3:** Comparison of the accuracy of *N*th order Markov model

Organism	# of genes	0th	1st	2nd
*E. coli*	858	92.4%	**93.2%**	92.7%
*B. subtilis*	1243	91.8%	**92.7%**	91.6%
*H. arsenicoxydans*	162	**92.6%**	90.1%	76.5%

#### Assimilation vs discrimination

To calculate the relevant Bayesian probability, we considered two alternative models (see Methods). In the first model, an assimilation model, we assumed that base frequencies of non-TIS sequences near a candidate start codons are the same as in the genome-wide background model (Eq. 8). In the second model, a discrimination model, we learned the base frequencies near a non-TIS from the negative data set (Eq. 9). This might have led to an improvement of the outcome, similar to that using discrimination in CDS identification, illustrated by the better accuracy using GeneMark in gene identification [2]. However, the overall accuracy reported by each model was exactly the same, although different genes were predicted incorrectly by the two approaches. This comparison shows that the differences between background and non-RBS sequences are relatively small.

In this paper, we used the assimilation model, as it is simpler than but achieves the same accuracy as the discrimination model.

#### Performance comparison

For *E. coli*, we correctly predicted 799/858 = 93.2% starts for the EcoGene data set and 184/191 = 96.3% for the Link data set [21]. For *B. subtilis*, 1152/1243 = 92.7% of TIS sites in the 'non-y' data set and 184/191 = 96.3% in an experimentally validated data set of 58 genes were predicted correctly. We compared the prediction of Hon-yaku's accuracy with that of other approaches: TiCo [7], MED-Start [22], and GS-Finder [23] (Table 4). To avoid overestimating the accuracies, we used the longest ORFs as input data instead of GenBank annotations, because some of our data sets are made from GenBank annotations with strong sequence homology to experimentally validated TIS from *E. coli *or *B. subtilis*. Another well known program, RBSfinder [24], appears to be extremely sensitive to the input TIS positions and the parameter for searching window size, making the comparison difficult. We listed the accuracy from the previous publication [22] for reference.

**Table 4:** Comparison with the TiCo, MED-Start, GS-Finder, and RBSfinder TIS prediction programs

Organism (data set)	# of genes	GC content	This method	TiCo^*a*^	MED-Start^*a*^	GS-Finder^*a*^	RBSfinder
*E. coli *(EcoGene)	858	50.8%	93.2%	**95.2%**	93.0%	91.1%	(81.9%^*b*^)
*E. coli *(Link)	191		96.3%	**96.9%**	**96.9%**	93.7%	(80.0%^*b*^)
*B. subtilis *(non-y)	1243	43.5%	**92.7%**	89.7%	91.2%	90.3%	(78.5%^*b*^)
*B. subtilis*	58		**96.6%**	91.4%	**96.6%**	**96.6%**	(82.8%^*b*^)
*P. aeruginosa*	347	66.6%	**92.8%**	90.5%	67.1%	91.1%	-
*B. pseudomallei*	238	68.1%	**89.9%**	86.6%	3.4%	87.8%	-
*H. arsenicoxydans*	162	54.3%	**92.6%**	-	87.7%	89.5%	-

^*a *^We used the longest ORFs as input data.

^*b *^The accuracies are from previously published results [22].

In contrast to a supervised learning method like Hon-yaku, these tools are sensitive to the input TIS annotation. TiCo and GS-Finder were more stable against the initial position compared to MED-Start and RBS finder. On the other hand, supervised methods depend on the quality and the size of their training set. To ensure the correct evaluation of our method, we also performed cross validation by randomly selecting 10% or 20% of the data sets as the validation set and training the program with the remainder, and repeated this procedure one thousand times (see Methods). The difference was < 0.5% in *E. coli *and *B. subtilis*, which have large data sets, and < 2% in other organisms with small data sets (Table 5). Except in the case of *E. coli*, we found a higher prediction accuracy with Hon-yaku as compared to existing methods. Interestingly, the accuracy in *E. coli *is higher than in *B. subtilis*, even though *B. subtilis *has a strong RBS sequence motif. This is presumably due to the widespread usage of translation initiation sites other than ATG in the latter. This may point to an unknown factor in the translation initiation machinery contributing to translation accuracy in Firmicutes, possibly related to the absence of an S1 protein in these organisms.

**Table 5:** Comparison with validation methods

Organism (data set)	# of genes	leave-one-out	10% cross validation	20% cross validation
*E. coli *(EcoGene)	858	93.2%	92.9%	92.7%
*B. subtilis *(non-y)	1243	92.7%	92.7%	92.4%
*P. aeruginosa*	347	92.8%	91.5%	90.8%
*B. pseudomallei*	238	89.8%	88.5%	88.0%
*H. arsenicoxydans*	162	92.6%	91.1%	91.0%

In Hon-yaku, the average distance between the true TIS and the predicted site is 26.2 codons for the 58 false predictions in *E. coli*.

#### Estimation of the minimum required size of the training data set

**Figure 1:**
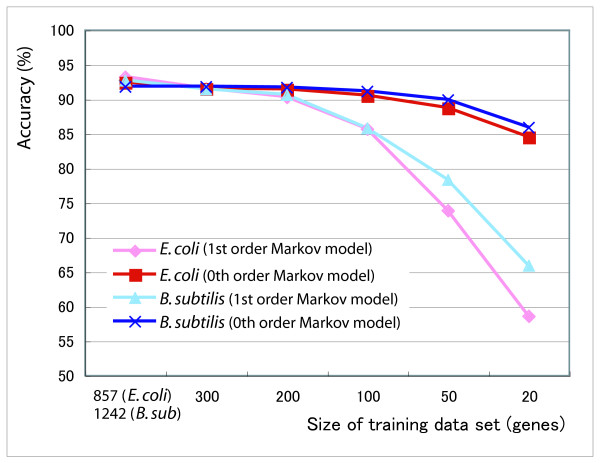
**Relationship between the size of training data set and the accuracy**. The x-axis shows the size of the training data set. The leftmost data point corresponds to the leave-one-out analysis based on the full data set of 857 genes in *E. coli *and 1242 genes in *B. subtilis*. For the other data points, we created the training data set of the given size by randomly selecting genes from the full data set.

The accuracy of supervised machine learning methods depends on the size of the training data set. To estimate the required minimum number of genes in the training data set, we calculated the prediction accuracy for different sizes of the training data set (Figure 1). When we trained Hon-yaku using 200 genes, the accuracy decreased by approximately three percent in both *E. coli *and *B. subtilis*. However, with first-order Markov model the accuracy decreased considerably when we trained with data sets consisting of less than 100 genes. For the zeroth-order Markov model, we found a small decrease.

### Genes without a canonical RBS motif

We analyzed the incorrect predictions for 58 genes in the data set of 858 *E. coli *genes. One main cause of incorrect predictions is the presence of a non-canonical RBS motif in the upstream sequence. To try and understand possible translation processes when a canonical RBS motif is not present, we considered the following three possibilities.

1. Split RBS motif, which would involve the S1 protein translation mechanism [25].

A RBS-like sequence is located in two separate positions in the upstream sequence of the S1 protein messenger RNA, which can fold into three consecutive hairpins. It was proposed that after a tertiary structure is created, both parts come next to each other and can act as a RBS sequence motif [25]. Recently, however, Skorski *et al*. showed that this was not the case, using ribosomes modified at the 3'end of their 16S RNA. They suggested that the GGA motifs present in the structured mRNA leader are recognized directly by the S1 protein and do not pair with the 16S RNA. S1 would then interact with the ribosome and properly position the mRNA for translation initiation [26]. Furthermore, for the 58 genes of interest, we predicted the secondary structure using Mfold [27]. Within the data set, we could not find convincing examples suggesting that they might generate a good RBS sequence after folding.

2. Leaderless mRNAs

Another possibility to translate mRNAs without RBS sequences is leaderless mRNA. Although computational methods to predict leaderless mRNAs are limited, we examined the assumption that a TIS is located at the very beginning of the transcribed sequence without a RBS sequence. We searched RNA polymerase recognition sequences using a published weight matrix for the major promoters in *E. coli *[28]. No clear motifs were detected in the region located approximately 10 bp upstream from the TIS. This supports the conjecture that leaderless mRNA is rather uncommon in Gram-negative bacteria [29]. Further experimental data are needed to explore whether this hypothesis is correct.

3. RBS-less translation supported by the S1 protein

In *E. coli*, the S1 protein assists in the unfolding of mRNA secondary structures and presentation to the ribosome. In contrast, *B. subtilis*, which does not have an S1 protein, is much less able to tolerate secondary structures in the translation initiation region. In vitro, S1 has no strict sequence specificity and binds polyU, polyC, and polyA as well as various heterogeneous RNAs, but it is involved in presenting particular structures to a virus-mediated RNA degradation pathway [30]. We therefore considered the possible role of secondary structure in the leader sequence of each mRNA coding for the unconventional CDSs. We applied Mfold to predict possible secondary structures and calculated the correlation with the strength of the RBS motif sequence. The correlation coefficient was 0.0195, showing that there was no correlation between the RBS motif intensity and the secondary structure thus predicted.

4. Known unconventional mRNA binding to 16S RNA. This has been demonstrated in the case of translation initiation factor IF3.

The TIS of *infC*, the structural gene for translational initiation factor IF3, starts with the unusual AUU codon both in *E. coli *[31] and *B. subtilis*, which are separated by 1.5 billion years of evolution.

The latest version of Colibri [32] contains four genes starting with ATT. We tried to predict these four genes by including a non-zero probability for an ATT start codon (see Methods). Only *infC *had a strong enough SD sequence to allow correct prediction against the small probability of an ATT start codon. Colibri has 37 genes with an atypical start codon, of which there are 28 kinds (other than NTG or ATT). Most of these genes code for a defective protein or are functionally unknown.

Presently Hon-yaku evaluates all ATG, GTG, and TTG codons in an ORF as candidate TISs. Hon-yaku can easily be extended to include other possible start codons. However, due to the low prior probability for atypical start codons, they can only be detected if preceded by a sufficiently strong SD sequence. Finally, several cases of spurious CDSs are created by the presence of codons for the 21st and 22nd amino acids, selenocysteine and pyrrolysine, coded by TGA and TAG codons respectively [33].

### Multi-TIS genes

The definition of a gene is notoriously difficult. In particular, it may happen that two different functional gene products are coded from the same DNA sequence, differing only in their start site. This is the case for the *B. subtilis lysC *gene, which codes for two proteins depending on two in frame start sites, resulting in a heterotetrameric alpha2/beta2 protein [34].

In the same way, both in *E. coli *and in *B. subtilis*, the gene *infB *codes for the two forms of the translational initiation factor IF2: IF2 alpha and IF2 beta. The *lacZ*::fused gene expresses two different products corresponding to the fused proteins IF2 alpha-beta-galactosidase and IF2 beta-beta-galactosidase, which confirms in vivo that the IF2 forms differ at their N terminus [35].

We presumed that some of the "false" predictions with a high Bayesian probability could be good candidates for genes that have two TISs (Table 6). We also checked the length difference and protein motifs for these cases to see whether the protein function would change upon change in start site. The Pfam [36] annotation did not point out particular domain structures that could be related to the difference in the TIS for any of the genes we identified. Nevertheless, we think that they might be good candidates for multiple authentic CDSs coded from a single ORF.

**Table 6:** Examples of candidate multi TISs predictions with a high Bayesian score

		Bayesian probability			
EG number	Gene	FP site*	TP site**	length difference	Pfam
EG10350	*fucK*	1.000	0.410	-10	-
EG10825	*recC*	0.932	0.866	-16	Exonuc_V_gamma
EG10106	*atpI*	0.851	0.099	+4	-
EG13547	*ykfE*	0.847	0.037	-9	-
EG10491	*iclR*	0.766	0.319	+11	-
EG10421	*guaB*	0.745	0.237	+23	-
EG11530	*fadD*	0.663	0.013	+11	-
EG10542	*lon*	0.621	0.471	-43	LON
EG10774	*prs*	0.522	0.011	+22	PsrA
EG10936	*secA*	0.515	0.073	-34	SecA

*FP: False positive predictions that have over 50% Bayesian probability.

**TP: True positive prediction with EcoGene data set.

Among incorrectly predicted genes, the Bayesian probability of an incorrect site was largest for the *fucK *gene. A BlastP search for counterparts in other genomes however suggested that the predicted start site is actually correct. Indeed, this putatively "false" TIS is annotated as the TIS in *Salmonella enterica *serovar Typhimurium LT2, *Yersinia bercovieri, Yersinia frederiksenii, Sodalis glossindius*, and *Shigella boydii*. We therefore presume that the Hon-yaku prediction is correct, and that the re-annotated *fucK *sequence is probably, for some reason, erroneous. Similar situations were uncovered in other genes, suggesting that the identification of the N-terminus of the corresponding proteins might not correspond to the primary translation product, but to some maturation product. Alternatively, those cases could suggest that some coding regions can code for polypeptides of different length, although a Pfam search did not reveal a salient functional difference between them. Finally, genes may keep multiple TIS candidates to gain robustness against gene mutations in the vicinity of the TIS.

## Conclusion

In an attempt to improve translation initiation site prediction and to make it applicable in a variety of bacterial genomes, we introduced biological knowledge of the translation process in the Hon-yaku algorithm. We considered the RBS sequence, the distance between the TIS and the RBS sequence, the nature of the start codon, the A-rich sequences following start codons, and the distribution of the protein length ratio to compute Bayesian joint score function. Additionally, using the operon structure predicted from the intergenic distances increases the accuracy by around 2%. Hon-yaku displays all these scores together with the total Bayesian probability for every TIS candidate as a means to improve the objectivity of human annotation.

In addition to user-friendliness, the reason why most existing programs adopt an unsupervised approach is the absence of experimentally validated TIS data. Although a supervised learning method requires more effort for the creation of a training data set, it identifies organism-specific features and allows the user to produce a final description of the best features relevant to a specific organism.

Hon-yaku uses a training set derived from models where TISs have been experimentally established (*E. coli *and *B. subtilis*), so strictly speaking, the extrapolating of our successful identifications are limited to Gamma-Proteobacteria and Firmicutes. Further work with other distant clades will be needed to see whether it can be generalised to the whole Bacteria kingdom.

## Methods

### Motif information content

Information content of motif *X *is

I(X)=∑iL(2+∑nP(xi,n)log2P(xi,n)),     (1)
 MathType@MTEF@5@5@+=feaafiart1ev1aaatCvAUfKttLearuWrP9MDH5MBPbIqV92AaeXatLxBI9gBaebbnrfifHhDYfgasaacH8akY=wiFfYdH8Gipec8Eeeu0xXdbba9frFj0=OqFfea0dXdd9vqai=hGuQ8kuc9pgc9s8qqaq=dirpe0xb9q8qiLsFr0=vr0=vr0dc8meaabaqaciaacaGaaeqabaqabeGadaaakeaacqWGjbqscqGGOaakcqWGybawcqGGPaqkcqGH9aqpdaaeWbqaaiabcIcaOiabikdaYiabgUcaRmaaqafabaGaemiuaaLaeiikaGIaemiEaG3aaSbaaSqaaiabdMgaPjabcYcaSiabd6gaUbqabaGccqGGPaqkieGacqWFSbaBcqWFVbWBcqWFNbWzdaWgaaWcbaGaeGOmaidabeaakiabdcfaqjabcIcaOiabdIha4naaBaaaleaacqWGPbqAcqGGSaalcqWGUbGBaeqaaOGaeiykaKIaeiykaKcaleaacqWGUbGBaeqaniabggHiLdaaleaacqWGPbqAaeaacqWGmbata0GaeyyeIuoakiabcYcaSiaaxMaacaWLjaWaaeWaaeaacqaIXaqmaiaawIcacaGLPaaaaaa@57C5@

where *i *is the position, *L *is the length of the motif, and *n *is the each nucleotide A, C, G, and T. For the information content calculation based on N data set sequences, we added N
 MathType@MTEF@5@5@+=feaafiart1ev1aaatCvAUfKttLearuWrP9MDH5MBPbIqV92AaeXatLxBI9gBaebbnrfifHhDYfgasaacH8akY=wiFfYdH8Gipec8Eeeu0xXdbba9frFj0=OqFfea0dXdd9vqai=hGuQ8kuc9pgc9s8qqaq=dirpe0xb9q8qiLsFr0=vr0=vr0dc8meaabaqaciaacaGaaeqabaqabeGadaaakeaadaGcaaqaaiabd6eaobWcbeaaaaa@2DEC@ pseudocounts, using the background probability of each base frequency. We used the upstream 30 bp and downstream 20 bp from TIS sites for the calculation.

### Experimentally validated data set for translation initiation sites

We used the EcoGene database [37] and Link data set [21] as reliable data sets of translation initiation sites in *E. coli*. The EcoGene database contains 862 proteins that were confirmed by N-terminal protein sequence identification. We removed from the data set a selenoprotein, release factor 2 (which is known to be synthesized by a + 1 frameshift), as well as two genes starting with ATT instead of canonical start codons (ATG, GTG, and TTG),.

The Link data set contains 195 genes; four of these are not consistent with the EcoGene data set. To construct a fully reliable data set, we removed these four genes (*hdeB, leuB, lolA*, and *ydcG*). For *B. subtilis*, we used a data set of 1248 'non-y' (i.e., experimentally characterized) genes [38] and checked them using the new GenBank annotation (NC_000964.2). Two genes had been removed in the new GenBank annotation, and three codons previously identified as start codons were changed to ATC, ATT, and CTG. We removed those data, leaving 1243 genes in the data set. We also included the more reliable 58 sequences confirmed by comparison with homologous sequences of *Bacillus halodurans *[38].

### Constructing data set with sequence homology

When we apply Hon-yaku to a newly sequenced bacterial genome such as *H. arsenicoxydans*, we need to construct a reliable data set with strong sequence homology to experimentally validated genes. Using the currently available two data sets, the EcoGene data set and the *B. subtilis *non-y data set, we defined presumably correct start sites for genomes where experimental data on actual start sites is missing by using the set of related persistent genes ([16], this works for Proteobacteria and Firmicutes) aligning them individually with counterparts in model organisms (*E. coli *and *B. subtilis*), and choosing manually the start site.

We substantiated the procedure by comparison with diverse *E. coli *and *B. subtilis *data sets as follows:

1. Pick up orthologous genes from the EcoGene data set or *B. subtilis *non-y data set.

We defined orthologous genes when two proteins display reciprocal best hit with at least 40% similarity in amino acid sequence and 20% or less difference in protein length [39]. We obtained 165 orthologous genes that belong to both the EcoGene data set and the *B. subtilis *non-y data set.

2. Remove genes that are not aligned in TIS vicinity or that have two or more candidate TISs within 5 bp. With the 165 orthologous genes, we confirmed that 89% of the TIS position differences are less than 5 bp. We removed genes whose TISs is not located within 5 bp upstream or downstream from the experimentally validated TIS, and that have no other candidate TIS within these 5 bp vicinity. From these rules, we obtained a data set of 126 genes with 100% accuracy out of the 165 orthologous genes.

We applied this procedure to *P. aeruginosa, B. pseudomallei*, and *H. arsenicoxydans *to construct the training data sets.

### Modeling to predict translation initiation sites

To construct a suitable score function, we applied Bayesian statistics to combine the following five elements:

1. The motif sequence around the ribosomal binding site (RBS), identifying the RBS region using a weight matrix constructed from the reference data set

2. The empirically determined distance between the RBS sequence and the start codon

3. The base composition of the start codon

4. The base composition of the beginning of the protein coding sequence with a position specific scoring matrix

5. The empirically determined length of the protein

Additionally we took into account overlapping ORFs using the empirically determined intergenic distance distributions. This methodology requires only the positions of stop codons and evaluates all TIS candidates that are located between the stop codon to the nearest upstream stop codon. We used the annotation by running GeneMark [2] on the genome of *H. arsenicoxydans *and by using GenBank entries for the other organisms.

#### Motif search around the RBS

One of the most important elements for TIS prediction is the RBS, containing the RBS sequence AGGAG in *E. coli *[8] and AAGGAGGU in *B. subtilis *[40].

Different tools adopted different methods to model the RBS. Hannenhalli *et al*. used the RBS binding energy to find the RBS motif [41]. The program RBSfinder considers the number of hydrogen bonds to detect motifs complementary to the 3' end of the 16S rRNA [24]. GS-Finder uses the "Z-curve" method [42], which considers differences of the cumulative occurrence numbers for three kinds of base combinations [23]. GS-Finder considers the A, C, G, T contexts in a window. Recently, because of the remarkable progress in motif extraction tools and to avoid having to calculate the binding energy between an organism-dependent 16S rRNA and the mRNA, position specific weight matrices (0th order Markov Model) have been applied for describing the RBS sequence motif (ex. MED-Start [22]). In this paper, we also used a zeroth-order Markov model, while, in addition, we explored higher-order Markov models. To describe the motif sequences by a 1st-order Markov model, we denote the transition probability of the double bases "mn" as *a*_*mn *_= *P *(*x*_*i *_= *n*|*x*_*x*-1 _= *m*). The probability that the motif sequence *S*_*M *_is generated by this model is then:

P(SM)=P(x1,x2,⋯,xL)=P(x1|x0)P(x2|x1)⋯P(xL|xL−1)ln⁡P(SM)=∑i=1Lln⁡(axi−1xi),     (2)
 MathType@MTEF@5@5@+=feaafiart1ev1aaatCvAUfKttLearuWrP9MDH5MBPbIqV92AaeXatLxBI9gBaebbnrfifHhDYfgasaacH8akY=wiFfYdH8Gipec8Eeeu0xXdbba9frFj0=OqFfea0dXdd9vqai=hGuQ8kuc9pgc9s8qqaq=dirpe0xb9q8qiLsFr0=vr0=vr0dc8meaabaqaciaacaGaaeqabaqabeGadaaakeaafaqaaeWadaaabaGaemiuaaLaeiikaGIaem4uam1aaSbaaSqaaiabd2eanbqabaGccqGGPaqkaeaacqGH9aqpaeaacqWGqbaucqGGOaakcqWG4baEdaWgaaWcbaGaeGymaedabeaakiabcYcaSiabdIha4naaBaaaleaacqaIYaGmaeqaaOGaeiilaWIaeS47IWKaeiilaWIaemiEaG3aaSbaaSqaaiabdYeambqabaGccqGGPaqkaeaaaeaacqGH9aqpaeaacqWGqbaucqGGOaakcqWG4baEdaWgaaWcbaGaeGymaedabeaakiabcYha8jabdIha4naaBaaaleaacqaIWaamaeqaaOGaeiykaKIaemiuaaLaeiikaGIaemiEaG3aaSbaaSqaaiabikdaYaqabaGccqGG8baFcqWG4baEdaWgaaWcbaGaeGymaedabeaakiabcMcaPiabl+UimjabdcfaqjabcIcaOiabdIha4naaBaaaleaacqWGmbataeqaaOGaeiiFaWNaemiEaG3aaSbaaSqaaiabdYeamjabgkHiTiabigdaXaqabaGccqGGPaqkaeaacyGGSbaBcqGGUbGBcqWGqbaucqGGOaakcqWGtbWudaWgaaWcbaGaemyta0eabeaakiabcMcaPaqaaiabg2da9aqaamaaqahabaGagiiBaWMaeiOBa4MaeiikaGIaemyyae2aaSbaaSqaaiabdIha4naaBaaameaacqWGPbqAcqGHsislcqaIXaqmaeqaaSGaemiEaG3aaSbaaWqaaiabdMgaPbqabaaaleqaaOGaeiykaKcaleaacqWGPbqAcqGH9aqpcqaIXaqmaeaacqWGmbata0GaeyyeIuoakiabcYcaSaaacaWLjaGaaCzcamaabmaabaGaeGOmaidacaGLOaGaayzkaaaaaa@871C@

where *i *is the position and *L *is the length of the motif.

The log-likelihood ratio that the sequence *S*_*M *_is created by the model is

M≡ln⁡P[SM|motif]P[SM|background]=∑i=1LWi,SM(xi−1xi),
 MathType@MTEF@5@5@+=feaafiart1ev1aaatCvAUfKttLearuWrP9MDH5MBPbIqV92AaeXatLxBI9gBaebbnrfifHhDYfgasaacH8akY=wiFfYdH8Gipec8Eeeu0xXdbba9frFj0=OqFfea0dXdd9vqai=hGuQ8kuc9pgc9s8qqaq=dirpe0xb9q8qiLsFr0=vr0=vr0dc8meaabaqaciaacaGaaeqabaqabeGadaaakeaacqWGnbqtcqGHHjIUcyGGSbaBcqGGUbGBdaWcaaqaaiabdcfaqjabcUfaBjabdofatnaaBaaaleaacqWGnbqtaeqaaOGaeiiFaWNaeeyBa0Maee4Ba8MaeeiDaqNaeeyAaKMaeeOzayMaeiyxa0fabaGaemiuaaLaei4waSLaem4uam1aaSbaaSqaaiabd2eanbqabaGccqGG8baFcqqGIbGycqqGHbqycqqGJbWycqqGRbWAcqqGNbWzcqqGYbGCcqqGVbWBcqqG1bqDcqqGUbGBcqqGKbazcqGGDbqxaaGaeyypa0ZaaabCaeaacqWGxbWvdaWgaaWcbaGaemyAaKMaeiilaWIaem4uam1aaSbaaWqaaiabd2eanbqabaWccqGGOaakcqWG4baEdaWgaaadbaGaemyAaKMaeyOeI0IaeGymaedabeaaliabdIha4naaBaaameaacqWGPbqAaeqaaSGaeiykaKcabeaaaeaacqWGPbqAcqGH9aqpcqaIXaqmaeaacqWGmbata0GaeyyeIuoakiabcYcaSaaa@6E9F@

where Wi,SM(mn)
 MathType@MTEF@5@5@+=feaafiart1ev1aaatCvAUfKttLearuWrP9MDH5MBPbIqV92AaeXatLxBI9gBaebbnrfifHhDYfgasaacH8akY=wiFfYdH8Gipec8Eeeu0xXdbba9frFj0=OqFfea0dXdd9vqai=hGuQ8kuc9pgc9s8qqaq=dirpe0xb9q8qiLsFr0=vr0=vr0dc8meaabaqaciaacaGaaeqabaqabeGadaaakeaacqWGxbWvdaWgaaWcbaGaemyAaKMaeiilaWIaem4uam1aaSbaaWqaaiabd2eanjabcIcaOiabd2gaTjabd6gaUjabcMcaPaqabaaaleqaaaaa@374E@ is the weight matrix of 1st-order Markov chain for a nucleotide *n *at position *i *to be followed by the nucleotide *m*. We prepared one log-odds scoring matrix *M*_*SD *_to describe the conserved region around the ribosomal binding site, and another matrix *M*_*DS *_to describe the downstream adenine-rich region following the start codon. Those motifs are defined by multiple alignments. In this section, we described the 1st order Markov model. When comparing the 0th, 1st, and 2nd order Markov model in *E. coli, B. subtilis*, and *Herminiimonas arsenicoxydans*, we found that a 1st-order Markov model yields more accurate results in both *E. coli *and *B. subtilis*, whereas a zeroth-order model was most accurate for *Herminiimonas arsenicoxydans *(Table 3).

#### The empirically determined distance from a RBS sequence to a start codon

**Figure 2:**
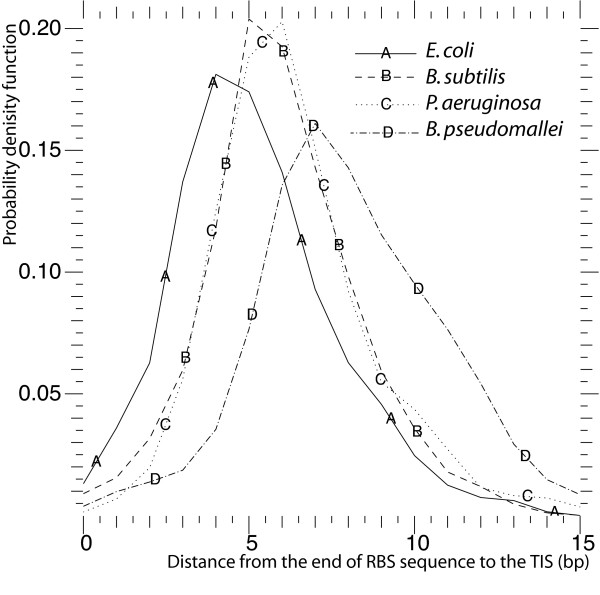
Distance distribution from the end of RBS sequence to the translation initiation sites.

To describe the gap length between a RBS sequence and a start codon, we estimated the probability density distributions *f*_dist _(*D*_*i*_) of the distance *D*_*i *_from the RBS sequence to the translation initiation site, measured in base pairs, using a kernel density estimation based on Gaussian kernels (Figure 2) [43]. The two Gram-negative bacteria, *E. coli *and *Herminiimonas arsenicoxydans*, have similar distributions of the length between the RBS sequence and the TIS, while the Gram-positive bacterium *B. subtilis *has a longer average distance between the RBS sequence and the start codon. This agrees with the results of previous reports [38,22].

#### Base composition of start codons

Table 1 shows the frequency of each start codon for the three bacteria. We also calculated the frequency of ATG, GTG, and TTG codons upstream and downstream of the true TIS to create a negative TIS data set (Eq. 9).

#### Distribution of protein length ratio

**Figure 3:**
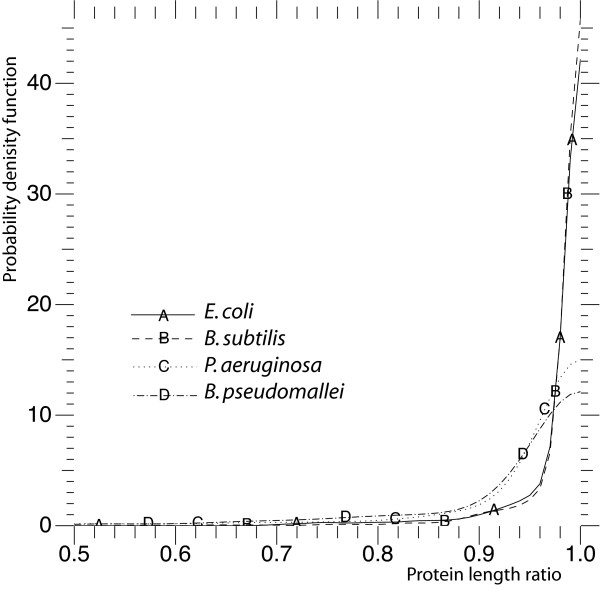
Distribution of protein length ratio.

62.6% of the EcoGene data set genes start with the first possible translation initiation codon as the real CDS. We also used the distribution of the ratio of the protein length to the length of the longest ORF. The smallest ratio is 0.697 in the EcoGene data set, most genes show a ratio of over 0.95 (Figure 3).

### Combining features around TIS

The Bayesian posterior probability that a gene starts from the translation initiation site *TIS *can be calculated as

P(TIS|S,Dprotein)=P(S,Dprotein|TIS)Pprior(TIS)∑P(S,Dprotein|TIS)Pprior(TIS),     (4)
 MathType@MTEF@5@5@+=feaafiart1ev1aaatCvAUfKttLearuWrP9MDH5MBPbIqV92AaeXatLxBI9gBaebbnrfifHhDYfgasaacH8akY=wiFfYdH8Gipec8Eeeu0xXdbba9frFj0=OqFfea0dXdd9vqai=hGuQ8kuc9pgc9s8qqaq=dirpe0xb9q8qiLsFr0=vr0=vr0dc8meaabaqaciaacaGaaeqabaqabeGadaaakeaacqWGqbaucqGGOaakcqWGubavcqWGjbqscqWGtbWucqGG8baFcqWGtbWucqGGSaalcqWGebardaWgaaWcbaGaemiCaaNaemOCaiNaem4Ba8MaemiDaqNaemyzauMaemyAaKMaemOBa4gabeaakiabcMcaPiabg2da9maalaaabaGaemiuaaLaeiikaGIaem4uamLaeiilaWIaemiraq0aaSbaaSqaaiabdchaWjabdkhaYjabd+gaVjabdsha0jabdwgaLjabdMgaPjabd6gaUbqabaGccqGG8baFcqWGubavcqWGjbqscqWGtbWucqGGPaqkcqWGqbaudaWgaaWcbaGaeeiCaaNaeeOCaiNaeeyAaKMaee4Ba8MaeeOCaihabeaakiabcIcaOiabdsfaujabdMeajjabdofatjabcMcaPaqaamaaqaeabaGaemiuaaLaeiikaGIaem4uamLaeiilaWIaemiraq0aaSbaaSqaaiabdchaWjabdkhaYjabd+gaVjabdsha0jabdwgaLjabdMgaPjabd6gaUbqabaGccqGG8baFcqWGubavcqWGjbqscqWGtbWucqGGPaqkcqWGqbaudaWgaaWcbaGaeeiCaaNaeeOCaiNaeeyAaKMaee4Ba8MaeeOCaihabeaakiabcIcaOiabdsfaujabdMeajjabdofatjabcMcaPaWcbeqab0GaeyyeIuoaaaGccqGGSaalcaWLjaGaaCzcamaabmaabaGaeGinaqdacaGLOaGaayzkaaaaaa@8E58@

where the prior probability *P*_prior _(*TIS*) is calculated as the frequency of start codon. *P *(*S, D*_*protein*_|*TIS*) is the conditional probability that the sequence *S *is generated around a true translation initiation site, resulting in a protein coding region of length *D*_*protein*_. The sequence *S *around the TIS consists of the ribosomal binding site *S*_*SD*_, the start codon *S*_*STC*_, the sequence *S*_*DS *_content downstream of the TIS, and the remaining sequence *S*\*S*_*SD *_*S*_*TIS *_*S*_*DS*_. We can then decompose *P *(*S, D*_*protein*_|*TIS*) into six parts:

*P *(*S, D*_*protein*_|*TIS*)

= *P *(*S*_*SD*_|*TIS*)·*f*_dist _(*D*_*SD*2*STC*_)·*P *(*S*_*STC*_|*TIS*)

·*P *(*S*_*DS*_|*TIS*)·*f*_dist _(*D*_*protein*_)·*P *(*S*\*S*_*SD *_*S*_*TIS *_*S*_*DS*_|background),     (5)

*f*_dist _(*D*_*SD*2*STC*_) is the probability that *S*_*RSB *_is generated at a distance *D*_*SD*2*STC *_from the transcription start site, and *f*_dist _(*D*_*protein*_) is the distribution of the protein length.

Dividing by the background probability yields

P(S,Dprotein|TIS)P(S,Dprotein|background)=eMSDfdist(DSD2STC)P(STC|TSS)eMDSfdist(Dprotein),     (6)
 MathType@MTEF@5@5@+=feaafiart1ev1aaatCvAUfKttLearuWrP9MDH5MBPbIqV92AaeXatLxBI9gBaebbnrfifHhDYfgasaacH8akY=wiFfYdH8Gipec8Eeeu0xXdbba9frFj0=OqFfea0dXdd9vqai=hGuQ8kuc9pgc9s8qqaq=dirpe0xb9q8qiLsFr0=vr0=vr0dc8meaabaqaciaacaGaaeqabaqabeGadaaakeaafaqadeGabaaabaWaaSaaaeaacqWGqbaucqGGOaakcqWGtbWucqGGSaalcqWGebardaWgaaWcbaGaemiCaaNaemOCaiNaem4Ba8MaemiDaqNaemyzauMaemyAaKMaemOBa4gabeaakiabcYha8jabdsfaujabdMeajjabdofatjabcMcaPaqaaiabdcfaqjabcIcaOiabdofatjabcYcaSiabdseaenaaBaaaleaacqWGWbaCcqWGYbGCcqWGVbWBcqWG0baDcqWGLbqzcqWGPbqAcqWGUbGBaeqaaOGaeiiFaWNaeeOyaiMaeeyyaeMaee4yamMaee4AaSMaee4zaCMaeeOCaiNaee4Ba8MaeeyDauNaeeOBa4MaeeizaqMaeiykaKcaaaqaaiabg2da9iabdwgaLnaaCaaaleqabaGaemyta00aaSbaaWqaaiabdofatjabdseaebqabaaaaOGaemOzay2aaSbaaSqaaiabbsgaKjabbMgaPjabbohaZjabbsha0bqabaGccqGGOaakcqWGebardaWgaaWcbaGaem4uamLaemiraqKaeGOmaiJaem4uamLaemivaqLaem4qameabeaakiabcMcaPiabdcfaqjabcIcaOiabdofatjabdsfaujabdoeadjabcYha8jabdsfaujabdofatjabdofatjabcMcaPiabdwgaLnaaCaaaleqabaGaemyta00aaSbaaWqaaiabdseaejabdofatbqabaaaaOGaemOzay2aaSbaaSqaaiabbsgaKjabbMgaPjabbohaZjabbsha0bqabaGccqGGOaakcqWGebardaWgaaWcbaGaemiCaaNaemOCaiNaem4Ba8MaemiDaqNaemyzauMaemyAaKMaemOBa4gabeaakiabcMcaPiabcYcaSaaacaWLjaGaaCzcamaabmaabaGaeGOnaydacaGLOaGaayzkaaaaaa@A05C@

where *M*_*SD *_and *M*_*DS *_are the value of the PSSM score for the RBS sequence and downstream region around the translation initiation site and *P *(*STC*|*TSS*) is the base composition of start codon, as determined from the *E. coli *known data set.

We define the score functions

score(*TIS*) ≡ ln *P*_prior _(*TIS*) + *M*_*SD *_+ ln *f*_dist _(*D*_*SD*2*STC*_)

+ ln *P *(*STC*|*TSS*) + *M*_*DS *_+ ln *f*_dist _(*D*_*protein*_).     (7)

For the calculation of *P *(*TIS*|*S, D*_*protein*_), we can consider either an assimilation method(Eq: 8) or a discrimination method(Eq: 9). The assimilation method makes the assumption that the base frequency around an ATG, GTG, TTG codon that is not a start codon is the same as the whole genome background model.

P(TIS|S)=P(S|TIS)Pprior(TIS)∑{true,neg}P(S|TIS)Pprior(TIS)=P(S|TIS)Pprior(TIS)P(S|TIS)Pprior(TIS)+P(S|nonTIS)Pprior(nonTIS)=escore(TIS)escore(TIS)+Pprior(nonTIS)     (8)
 MathType@MTEF@5@5@+=feaafiart1ev1aaatCvAUfKttLearuWrP9MDH5MBPbIqV92AaeXatLxBI9gBaebbnrfifHhDYfgasaacH8akY=wiFfYdH8Gipec8Eeeu0xXdbba9frFj0=OqFfea0dXdd9vqai=hGuQ8kuc9pgc9s8qqaq=dirpe0xb9q8qiLsFr0=vr0=vr0dc8meaabaqaciaacaGaaeqabaqabeGadaaakeaafaqaaeWadaaabaGaemiuaaLaeiikaGIaemivaqLaemysaKKaem4uamLaeiiFaWNaem4uamLaeiykaKcabaGaeyypa0dabaWaaSaaaeaacqWGqbaucqGGOaakcqWGtbWucqGG8baFcqWGubavcqWGjbqscqWGtbWucqGGPaqkcqWGqbaudaWgaaWcbaGaeeiCaaNaeeOCaiNaeeyAaKMaee4Ba8MaeeOCaihabeaakiabcIcaOiabdsfaujabdMeajjabdofatjabcMcaPaqaamaaqababaGaemiuaaLaeiikaGIaem4uamLaeiiFaWNaemivaqLaemysaKKaem4uamLaeiykaKIaemiuaa1aaSbaaSqaaiabbchaWjabbkhaYjabbMgaPjabb+gaVjabbkhaYbqabaGccqGGOaakcqWGubavcqWGjbqscqWGtbWucqGGPaqkaSqaaiabcUha7jabdsha0jabdkhaYjabdwha1jabdwgaLjabcYcaSiabd6gaUjabdwgaLjabdEgaNjabc2ha9bqab0GaeyyeIuoaaaaakeaaaeaacqGH9aqpaeaadaWcaaqaaiabdcfaqjabcIcaOiabdofatjabcYha8jabdsfaujabdMeajjabdofatjabcMcaPiabdcfaqnaaBaaaleaacqqGWbaCcqqGYbGCcqqGPbqAcqqGVbWBcqqGYbGCaeqaaOGaeiikaGIaemivaqLaemysaKKaem4uamLaeiykaKcabaGaemiuaaLaeiikaGIaem4uamLaeiiFaWNaemivaqLaemysaKKaem4uamLaeiykaKIaemiuaa1aaSbaaSqaaiabbchaWjabbkhaYjabbMgaPjabb+gaVjabbkhaYbqabaGccqGGOaakcqWGubavcqWGjbqscqWGtbWucqGGPaqkcqGHRaWkcqWGqbaucqGGOaakcqWGtbWucqGG8baFcqWGUbGBcqWGVbWBcqWGUbGBcqWGubavcqWGjbqscqWGtbWucqGGPaqkcqWGqbaudaWgaaWcbaGaeeiCaaNaeeOCaiNaeeyAaKMaee4Ba8MaeeOCaihabeaakiabcIcaOiabd6gaUjabd+gaVjabd6gaUjabdsfaujabdMeajjabdofatjabcMcaPaaaaeaaaeaacqGH9aqpaeaadaWcaaqaaiabdwgaLnaaCaaaleqabaGaee4CamNaee4yamMaee4Ba8MaeeOCaiNaeeyzauMaeiikaGIaemivaqLaemysaKKaem4uamLaeiykaKcaaaGcbaGaemyzau2aaWbaaSqabeaacqqGZbWCcqqGJbWycqqGVbWBcqqGYbGCcqqGLbqzcqGGOaakcqWGubavcqWGjbqscqWGtbWucqGGPaqkaaGccqGHRaWkcqWGqbaudaWgaaWcbaGaeeiCaaNaeeOCaiNaeeyAaKMaee4Ba8MaeeOCaihabeaakiabcIcaOiabd6gaUjabd+gaVjabd6gaUjabdsfaujabdMeajjabdofatjabcMcaPaaaaaGaaCzcaiaaxMaadaqadaqaaiabiIda4aGaayjkaiaawMcaaaaa@F412@

where *nonTIS *represents an ATG, GTG, or TTG codon that does not function as a start codon.

In the discrimination method, we need to make negative data sets which explicitly model *nonTIS *features. In this case, we made two models, which represent the upstream (intergenic) region *nonTIS*_*up*_, and the downstream (in coding region) *nonTIS*_*down *_to distinguish between protein coding features and non-coding features.

P(TIS|S)=P(S|TIS)Pprior(TIS)∑{true,negup,negdown}P(S|TIS)Pprior(TIS)=escore(TIS)escore(TIS)+escore(nonTISup)+escore(nonTISdown)     (9)
 MathType@MTEF@5@5@+=feaafiart1ev1aaatCvAUfKttLearuWrP9MDH5MBPbIqV92AaeXatLxBI9gBaebbnrfifHhDYfgasaacH8akY=wiFfYdH8Gipec8Eeeu0xXdbba9frFj0=OqFfea0dXdd9vqai=hGuQ8kuc9pgc9s8qqaq=dirpe0xb9q8qiLsFr0=vr0=vr0dc8meaabaqaciaacaGaaeqabaqabeGadaaakeaafaqaaeGadaaabaGaemiuaaLaeiikaGIaemivaqLaemysaKKaem4uamLaeiiFaWNaem4uamLaeiykaKcabaGaeyypa0dabaWaaSaaaeaacqWGqbaucqGGOaakcqWGtbWucqGG8baFcqWGubavcqWGjbqscqWGtbWucqGGPaqkcqWGqbaudaWgaaWcbaGaeeiCaaNaeeOCaiNaeeyAaKMaee4Ba8MaeeOCaihabeaakiabcIcaOiabdsfaujabdMeajjabdofatjabcMcaPaqaamaaqababaGaemiuaaLaeiikaGIaem4uamLaeiiFaWNaemivaqLaemysaKKaem4uamLaeiykaKIaemiuaa1aaSbaaSqaaiabbchaWjabbkhaYjabbMgaPjabb+gaVjabbkhaYbqabaGccqGGOaakcqWGubavcqWGjbqscqWGtbWucqGGPaqkaSqaaiabcUha7jabdsha0jabdkhaYjabdwha1jabdwgaLjabcYcaSiabd6gaUjabdwgaLjabdEgaNnaaBaaameaacqWG1bqDcqWGWbaCaeqaaSGaeiilaWIaemOBa4MaemyzauMaem4zaC2aaSbaaWqaaiabdsgaKjabd+gaVjabdEha3jabd6gaUbqabaWccqGG9bqFaeqaniabggHiLdaaaaGcbaaabaGaeyypa0dabaWaaSaaaeaacqWGLbqzdaahaaWcbeqaaiabbohaZjabbogaJjabb+gaVjabbkhaYjabbwgaLjabcIcaOiabdsfaujabdMeajjabdofatjabcMcaPaaaaOqaaiabdwgaLnaaCaaaleqabaGaee4CamNaee4yamMaee4Ba8MaeeOCaiNaeeyzauMaeiikaGIaemivaqLaemysaKKaem4uamLaeiykaKcaaOGaey4kaSIaemyzau2aaWbaaSqabeaacqqGZbWCcqqGJbWycqqGVbWBcqqGYbGCcqqGLbqzcqGGOaakcqWGUbGBcqWGVbWBcqWGUbGBcqWGubavcqWGjbqscqWGtbWudaWgaaadbaGaemyDauNaemiCaahabeaaliabcMcaPaaakiabgUcaRiabdwgaLnaaCaaaleqabaGaee4CamNaee4yamMaee4Ba8MaeeOCaiNaeeyzauMaeiikaGIaemOBa4Maem4Ba8MaemOBa4MaemivaqLaemysaKKaem4uam1aaSbaaWqaaiabdsgaKjabd+gaVjabdEha3jabd6gaUbqabaWccqGGPaqkaaaaaaaakiaaxMaacaWLjaWaaeWaaeaacqaI5aqoaiaawIcacaGLPaaaaaa@CF71@

In Hon-yaku, we calculate *score *(*TIS*) and the Bayesian posterior probability that a gene starts from the TIS for all translation initiation sites in the ORF.

### Other contributing elements

To increase the prediction accuracy, we additionally considered the operon structure, and alternative candidate start codons that are either adjacent or separated by one codon.

If the two genes are arranged in a head-to-head configuration and the intergenic distance is under 100 bp, we added an empirically determined intergenic distance distribution ln (*f*_*dist *_(*D*_*headtohead*_)) to the score function (Eq. 7). If the two genes have the same direction and the intergenic distance is under 50 bp, we added an empirically determined intergenic distance distribution ln (*f*_*dist *_(*D*_*tailtohead_under*50*bp*_)) to the score function. Thus, we aimed to reduce mispredictions leading to genes with long overlapping sequence regions. This function also improves the prediction of genes with the start codon close to the previous stop codon, as often occurs in operons.

Another reason for incorrect predictions is that some genes have two start codon candidates close to each other. Especially when two candidates are contiguous, the distance function between the start codon and the RBS sequence *f*_*dist *_(*D*_*SD*2*STC*_) gives ambiguous results. In this case, our algorithm chooses the TIS based on the distribution of the start codon location for MM and MXM amino acid sequences. We constructed the species-specific distribution in *E. coli *and *B. subtilis *and applied the *E. coli *distribution to other bacteria that have a small number of data set genes.

Except for this two neighboring start codon case, which had to be fixed as described above, we established the value of all other parameters using the training data set.

### Cross validation

In this paper, we calculated accuracies of Hon-yaku with a leave-one-out cross validation analysis. To avoid showing only the overoptimistic performance rates of the leave-one-out measure, we also calculated the performance of our method with other cross validations. We trained our model with 90% or 80% of the true data set, while the randomly chosen remaining 10% or 20% are retained for subsequent use in evaluating our model. The procedure was repeated one thousand times.

## Authors' contributions

YM designed the algorithm and performed the study. MdH contributed the methological discussion. AD proposed the rationale for the study and outlined its biological implications. All authors participated in the writing of this article.
